# Prévalence des dyslipidémies au laboratoire de biochimie du CHU Aristide le Dantec de Dakar, Sénégal

**DOI:** 10.11604/pamj.2016.25.67.7758

**Published:** 2016-10-03

**Authors:** Fatou Cissé, Fatou Diallo Agne, Alassane Diatta, Abdou Salam Mbengue, Arame Ndiaye, Abdourahmane Samba, Souleymane Thiam, Dominique Doupa, Gaston Ndéné Sarr, Niama Diop Sall, Méissa Touré

**Affiliations:** 1 Laboratoire de Biochimie et Biologie Moléculaire, Faculté de Médecine, de Pharmacie et d’Odontostomatologie, Université Cheikh Anta Diop de Dakar, Sénégal; 2 Université Assane SECK de Ziguinchor, Sénégal; 3 Laboratoire de Bactériologie-Virologie, Hôpital Aristide Le Dantec, Dakar, Sénégal; 4 Laboratoire de Biochimie-Biologie Moléculaire Unité de Formation et de Recherche (UFR), Santé Université Gaston Berger, Saint Louis, Sénégal

**Keywords:** Dyslipidémie, prévalence, Sénégal, Dyslipidemia, prevalence, Senegal

## Abstract

**Introduction:**

L'objectif de cette étude était d'évaluer la prévalence des dyslipidémies chez les patients reçus au laboratoire de Biochimie de l'Hôpital Aristide Le Dantec pour le dosage d'un paramètre lipidique au cours de l'année 2013.

**Méthodes:**

Il s'agit d'une étude rétrospective portant sur 1356 patients âgés de 10 à 94 ans reçus au laboratoire de Biochimie du CHU Le Dantec de janvier à décembre 2013. Etaient inclus dans l'étude, tous les patients ayant au moins un paramètre du bilan lipidique dont les résultats étaient enregistrés dans le registre du laboratoire. Le cholestérol total, le cholestérol HDL, le cholestérol LDL ainsi que les triglycérides ont été dosés grâce à des méthodes enzymatiques sur un automate de Biochimie de type Cobas Integra 400 (Roche Diagnostics).

**Résultats:**

La prévalence des dyslipidémies dans notre population d'étude est de 39,30%. Les prévalences de l'hypercholestérolémie, l'hypoHDLémie, l'hyperLDLémie, l'hypertriglycéridémie et l'hyperlipidémie mixte étaient respectivement : 30,89% ; 7,30% ; 31,19% ; 0,51% ; 7,22%. Les sujets de 40 à 59 ans semblaient être plus exposés et on note une prédominance féminine en ce qui concerne l'hypercholestérolémie (54,17% vs 45,82%), l'hypoHDLémie (54,54% vs45, 45%), et l'hyperlipidémie mixte (51,08% vs 48,97%). Enfin les dyslipidémies étaient fortement corrélées à l'HTA et l'obésité.

**Conclusion:**

La forte prévalence des dyslipidémies retrouvée dans notre étude démontre l'intérêt d'étudier la prévalence des facteurs de risque cardio-vasculaires en particulier les dyslipidémies dans la population sénégalaise.

## Abstract (en)

**Introduction:**

This study aims to evaluate the prevalence of dyslipidemias among patients who underwent assay of a lipid parameter at the Laboratory of Biochemistry of Aristide Le Dantec University Hospital in 2013.

**Methods:**

We conducted a retrospective study of 1356 patients between ages 10-94 years presenting at the laboratory of Biochemistry of CHU Le Dantec from January to December 2013. All patients who had undergone at least one lipid assay whose results were recorded in the laboratory register were enrolled in the study. Total cholesterol, HDL cholesterol, LDL cholesterol and triglycerides were measured using enzymatic methods (Cobas Integra 400 plus biochemistry analyzer - Roche Diagnostics).

**Results:**

The prevalence of dyslipidemia in our study population was 39.30%. The prevalence of hypercholesterolemia, hypo-HDL-cholesterolemia, hyper-HDL-cholesterolemia, hypertriglyceridemia, mixed hyperlipidemia was 30,89% ; 7,30% ; 31,19% ; 0,51% ; 7,22% respectively. The subjects aged 40 to 59 years appeared to be more exposed. Moreover, there was a female predominance in hypercholesterolemia (54.17% vs 45.82%) the hypocholesterolemia (54.54% vs45,45%), and mixed hyperlipidemia (51.08% vs 48.97%). Dyslipidemias were strongly correlated to hypertension and obesity.

**Conclusion:**

The high prevalence of dyslipidemias found in our study highlights the importance of studying the prevalence of cardiovascular risk factors, in particular dyslipidemias, in Senegalese population.

## Introduction

Les dyslipidémies représentent un réel problème de santé publique avec des prévalences qui dépassent 30% dans les pays occidentaux [1–3]. En Afrique subsaharienne les prévalences varient selon la région et des taux de plus de 50% ont été retrouvés au Ghana [4] et au Nigéria [5]. Au Sénégal, malgré la fréquence des maladies cardio-vasculaires, les données sur la prévalence des facteurs de risque sont rares. Seules quelques enquêtes épidémiologiques ont été réalisées dans la ville de St Louis [6, 7] et en zone rurale (Guéoul) [8]. La recherche de ces facteurs de risque et leur prise en charge adéquate pourrait contribuer à prévenir les maladies cardio-vasculaires. Le bilan lipidique, examen simple et accessible à tous les laboratoires est une étape dans cette stratégie de prévention. Ainsi dans ce travail, nous nous sommes fixés comme objectif d'évaluer la prévalence des dyslipidémies chez le patients reçus au laboratoire de Biochimie de l'Hopital Aristide le Dantec pour le dosage d'un paramètre lipidique au cours de l'année 2013.

## Méthodes

Il s'agit d'une étude rétrospective portant sur 1356 patients âgés de 10 à 94 ans reçus au laboratoire de Biochimie du CHU Arisitide Le Dantec de janvier à décembre 2013. Etaient inclus dans l'étude, tous les patients ayant au moins un paramètre du bilan lipidique. Le cholestérol total, le cholestérol HDL, le cholestérol LDL ainsi que les triglycérides ont été dosés grâce à des méthodes enzymatiques sur l'automate de Biochimie Cobas Integra 400 (Roche Diagnostics). Les dyslipidémies ont été définies selon les critères du NCEP (The National Cholesterol Education Program) [9]. -hypercholestérolémie (cholestérol total >2g/l) -hypoHDLémie (cholestérol HDL <0,4g/l) -hypertriglycéridémie (triglycérides > 1,5g/l) - hyperlipidémie mixte (cholestérol total >2g/l et triglycérides > 1,5g/l) -hyperLDLémie(cholestérol LDL>1,3g/l) Les données ont été collectées sur Excell 2007 et analysées par le logiciel Epi Info 7(version 7.1.0, CDC Atlanta). Les tests statistiques ont été effectués en utilisant le test de khi-deux avec un seuil de significativité de 0,05.

## Résultats

**Figure 1:**
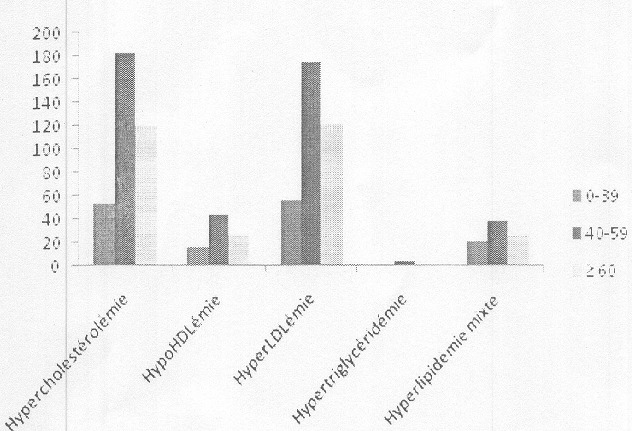
Distribution des dyslipidémies en fonction de l’âge

**Figure 2:**
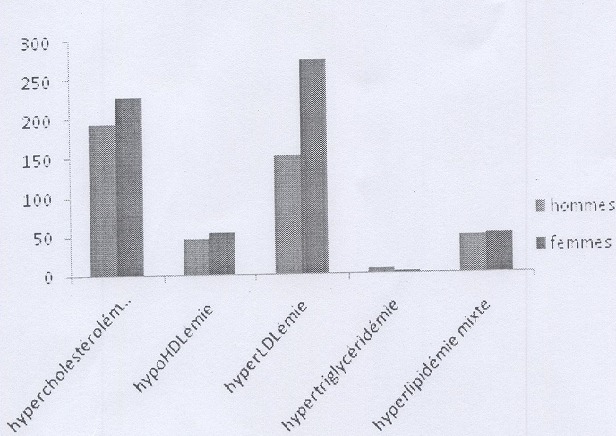
Répartition des dyslipidémies en fonction du sexe

Le Tableau 1 résume les caractéristiques de la population d'étude. On note une prédominance féminine avec un sex ratio de 0,76. La tranche d'âge 40-59 est la plus représentée aussi bien chez les hommes que chez les femmes. 47,12% des sujets étaient hospitalisés au niveau des différents services de l'hôpital. Seuls 45% des bulletins d'analyse comportaient une information clinique et parmi les diagnostics notés, l'HTA était en tête de fil suivi des complications cardio-vasculaires. La prévalence des dyslipidémies dans notre population d'étude était de 39,30%. Les prévalences de l'hypercholestérolémie, l'hypoHDLémie, l'hyperLDLémie, l'hypertriglycéridémie et l'hyperlipidémie mixte étaient respectivement : 30,89% ; 7,30% ; 31,19% ; 0,51% ; 7,22% (Tableau 2). La répartition des dyslipidémies en fonction de l'âge (Figure 1), montre que les sujets de 40 à 59 ans sont plus exposés. L'étude de la relation entre les dyslipidémies et le sexe (Figure 2) a montré une prédominance féminine en ce qui concerne l'hypercholestérolémie et l'hyperLDLémie (p<0,01) L'analyse multivariée entre la variable dyslipidémie et les autres facteurs de risque cardio-vasculaire (Tableau 3) montrent une association entre les dyslipidémies et d'une part l'HTA (RR=1,26) et d'autre part l'obésité (RR= 1,94).

**Tableau 1:** Caractéristiques sociodémographiques de la population d’étude

Variables	Total n (%)	Dyslipidémie	
		Oui n (%)	Non n (%)
**Sexe**			
Masculin	588(43,36)	194(32,99)	394(67)
Féminin	768(56,63)	339(44,14)	429(55,85)
**Sex ratio**	0,76		
**Age (années)**			
0-39	266(24,33)	77(28,94)	189(71,05)
40-59	438(40,07)	229(52,28)	209(47,71)
≥60	389(35,59)	151(38,81)	238(61,18)
**Provenance**			
Hospitalisés	639(47,12)	135(21,12)	504(78,87)
Externes	717(52,87)	398(55,5)	319(444,9)
**Diagnostic clinique**			
HTA	282(20,79)	136(48,22)	146(517,7
Diabète	33(2,43)	13(39,39)	20(60,6)
Obésité	11(0,81)	9(81,81)	2(18,18)
Complications cardio-vasculaires	73(5,38)	38(52,05)	35(47,94)
Syndrome néphrotique	11(0,81)	11(100)	0(0)
Autres	202(14,89)	55(27,22)	147(72,77)
Absence d'information clinique	744(54,86)	271(36,42)	473(63,57)

**Tableau 2:** Prévalence des dyslipidémies

Prévalence	Effectif	Pourcentage (%)
Dyslipidémies	533	39,30
Hypercholestérolémie	419	30,89
hypoHDLémie	99	7,30
hyperLDLémie	423	31,19
Hypertriglycéridémie	7	0,51
Hyperlipidémie mixte	98	7,22

**Tableau 3:** Corrélation entre dyslipidémies et autres facteurs de risque cardio-vasculaire (HTA, diabète, obésité)

Dyslipidémies			
	oui	non	RR
**HTA**			
Oui (n=282)	136	146	1,26
Non (n=330)	126	204	
**Diabète**			
Oui (n=33)	13	20	0,91
Non (n=579)	249	330	
**Obésité**			
Oui ( n=11)	9	2	1,94
Non( n=601)	253	348	

## Discussion

Le rôle majeur des dyslipidémies dans la genèse des maladies cardiovasculaires a été établi par de grandes études réalisées dans des cohortes de population notamment celle de Framingham aux États-Unis [10] et l'étude PROCAM en Europe [11]. Ce travail dont l'objectif était d'évaluer la prévalence des dyslipidémies chez les patients reçus au laboratoire de Biochimie de L'Hôpital Aristide Le Dantec pour un bilan lipidique a retrouvé un taux élevé (39,30%) (Tableau 2). Ces chiffres sont superposables à ceux retrouvés par Tiahou et ses collaborateurs [12] qui ont effectué une étude similaire au CHU de Cocody en Côte d'Ivoire. Ils corroborent aussi les prévalences retrouvées dans des enquêtes épidémiologiques portant sur les facteurs de risque cardio-vasculaires réalisées à St Louis [7] et à Guéoul [8]. Nos résultats dépassent largement ceux retrouvés en Algerie (14,3%) [13] et en Mauritanie (>14,8%) [14] et se rapprochent des prévalences observées dans les pays industrialisés qui dépassent 30% [1–3]. L'hyperLDLémie est la plus fréquente des dyslipidémies (31,19%) suivie de près par l'hypercholestérolémie (30,89%) (Tableau 2). Cette prédominance de l'hyperLDLémie a été aussi rapportée par les travaux de Doupa et al à St Louis [6] ainsi que Erem et al en Turquie [15]. Cependant la plupart des auteurs ont retrouvé une prédominance de l'hypercholestérolémie [2, 4, 12, 16–18]. Ceci pourrait être expliqué par le fait que ces auteurs n'ont pas tenu en considération, l'hyperLDLémie presque toujours associée à l'hypercholestérolémie. En effet 98% de nos sujets qui avaient une hypercholestéolémie présentaient aussi une hyperLDLémie. Concernant l'âge, notre étude a montré que les sujets de 39 à 59 étaient les plus touchés (Figure 1). Ces données sont confirmées par l'Etude Nationale-Nutriton Santé réalisée en France en 2006 qui a retrouvé une prévalence des dyslipidémies d'environ 67 % dans la classe d'âge des sujets de 55 à 74 ans, Gao Y. et ses collaborateurs ont aussi retrouvé une augmentation de l'incidence des dyslipidémies avec l'âge chez des travailleurs d'une entreprise chinoise [19]. Les femmes semblent être plus exposées à l'hypercholestérolémie (Figure 2). En effet, la plupart des études ont retrouvé une prédominance des dyslipidémies et plus particulièrement de l'hypercholestérolémie chez les femmes [2, 6, 7, 16]. Bien que l'hypercholestérolémie et son corrolaire l'hyperLDLémie semblent être les préoccupations majeures, les autres types de dyslipidémies devraient être pris en compte. Dans notre étude les prévalences de l'hypoHDLémie, de l'hyperlipidémie mixte, et de l'hypertriglycéridémie étaient respectivement 7,30%, 7,22% et 0,51%. Cet ordre de fréquence est aussi rapporté par les résultats publiés en 2005 par l'étude MONICA [20]. De même dans l'étude de Ferrieres J. et al [1], l'hypoHDLémie était la 2^ème^ anomalie lipidique la plus fréquente. Néanmoins, des différences ont été observées selon les auteurs. Sur le plan clinique, seuls 45% de nos patients avaient un diagnostic évident (Tableau 1), ceci démontre le manque d'information sur les patients dont les biologistes font souvent face. L'exploitation de ces données nous a permis de retrouver une association entre la dyslipidémie et les autres facteurs de risque de risque tels que l'HTA (RR=1,26) et l'obésité (RR=1,94) (Tableau 3). Cette association a été aussi retrouvée dans l'étude de Pessinaba [7]. La présence de complications cardiovasculaires à type d'insuffisance cardiaque, de syndrome coronarien et d'accident vasculaire cérébrale ischémique chez 52,05% des patients dyslipidémiques démontre la nécessité d'une prise en charge adéquate de ces facteurs de risque.

## Conclusion

Cette étude met en évidence une forte prévalence des dyslipidémies chez les sujets reçus au laboratoire de Biochimie de l'Hôpital Aristide Le dantec. Et les résultats trouvés pourraient être sous-estimés dans la mesure où la plupart de ces patients étaient déjà en contact avec le système de santé et seraient peut être sous traitement hypocholestérolémiant. Ceci démontre l'intérêt de réaliser une enquête épidémiologique sur les facteurs de risque cardio-vasculaire au niveau national.

### Etat des connaissances actuelles sur le sujet

- Au Sénégal, malgré la fréquence des maladies cardio-vasculaires, les données sur la prévalence des facteurs de risque sont rares. Seules quelques enquêtes épidémiologiques ont été réalisées dans la ville de St Louis et en zone rurale.

### Contribution de notre étude à la connaissance

- Ce travail fournit des données sur la prévalence hospitalière des dyslipidémies à Dakar (Sénégal). Les chiffres retrouvés dans notre étude démontrent l'intérêt d'une enquête épidémiologique au niveau national.
